# The Fluorescent Palette of DNA-Templated Silver Nanoclusters for Biological Applications

**DOI:** 10.3389/fchem.2020.601621

**Published:** 2020-11-11

**Authors:** Min Yang, Xu Chen, Yuan Su, Haiyan Liu, Hongxing Zhang, Xiangyang Li, Wentao Xu

**Affiliations:** ^1^Beijing Laboratory of Food Quality and Safety, Beijing Key Laboratory of Agricultural Product Detection and Control of Spoilage Organisms and Pesticide Residue, Faculty of Food Science and Engineering, Beijing University of Agriculture, Beijing, China; ^2^Institute of Nutrition and Health, China Agricultural University, Beijing, China; ^3^Faculty of Public Health, North China University of Technology, Tangshan, China

**Keywords:** noble metal nanoclusters, fluorescent probing, sensing, imaging, DNA-templated silver nanoclusters

## Abstract

Recently years have witnessed a surge in application of DNA-AgNCs in optics, catalysis, sensing, and biomedicine. DNA-templated silver nanoclusters (DNA-AgNCs), as emerging fluorophores, display superior optical performance since their size is close to the Fermi wavelength. DNA-AgNCs possess unique features, including high fluorescence quantum yields and stability, biocompatibility, facile synthesis, and low toxicity, which are requisite for fluorescent probes. The fluorescent emission of DNA-AgNCs can cover the violet to near-infrared (NIR) region by varying the DNA sequences, lengths, and structures or by modifying the environmental factors (such as buffer, pH, metal ions, macromolecular polymers, and small molecules). In view of the above excellent properties, we overview the DNA-AgNCs from the viewpoints of synthesis and fluorescence properties, and summarized its biological applications of fluorescence sensing and imaging.

## Introduction

The optical and electronic properties of metals depend largely on their size, especially in the nano scale (Diez and Ras, 2011). Noble metal nanoclusters (M NCs) composed of several to a few hundred metal atoms with sizes ~2 nm, which approaches the Fermi wavelength of electrons leading to molecule-like properties, such as discrete energy levels, good photostability, biocompatibility, as well as size dependent photoluminescence (Jin et al., 2016; Chakraborty and Pradeep, 2017; Chen et al., 2018). Therefore, M NCs exhibited strong light absorption and emission due to the electronic transitions between different energy levels (Huang et al., 2019). If there is no stabilizing scaffold, the metal nanoclusters will interact strongly with each other and gather irreversibly, thereby reducing their surface energy. Compared with other scaffold, DNA is very suitable as a template for nanofabrication due to its relative physiological stability and pre-designable structure (Seeman, 2003; Wilner and Willner, 2012).

DNA-AgNCs, as a novel kind of fluorescent nanomaterial, was first reported by Petty (Petty et al., 2004). Compared with other emerging M NCs, such as DNA-templated copper nanoclusters, DNA-AgNCs show a more extensive range and more adjustable fluorescence wavelength, excellent stability, higher fluorescence quantum yields, and lower toxicity (Latorre and Somoza, 2012; Cao et al., 2019a; Xu et al., 2020a). For example, it has been reported that the fluorescence quantum yield (QY) of silver nanoclusters (AgNCs) prepared in DNA is as high as 64% (Sharma et al., 2010). In addition, significant DNA-AgNCs fluorescence can be retained for at least a year by using a rationally designed DNA sequence (Sharma et al., 2012). Compared with other luminescent nanomaterials such as semiconductor quantum dots, the overall size of fluorescent AgNCs is extremely small, which makes them attractive as fluorescent biomarkers because the biological processes may be minimally perturbed by AgNC-based labeling (Yu et al., 2008). Furthermore, nanoclusters can be used as sensitive probes because of their optical response highly dependent on the interaction with the DNA oligomers (Diez and Ras, 2011). Therefore, the photostability and biocompatibility of DNA-AgNCs make them more suitable for biological systems, and have broad application prospects in biological research. Up to now, many nano-biosensors have been constructed based on the fluorescence properties of DNA-AgNCs.

Herein, we overview the DNA-AgNCs from the viewpoints of synthesis and fluorescence properties. In particular, the key factors affecting the fluorescence emission are summarized for the detection of more targets via intelligent design. Recent advancement of DNA-AgNCs in biological applications is expounded from two aspects of fluorescence sensing and imaging. Finally, current challenges, the perspectives and future directions on the DNA-AgNCs based biosensing and imaging are envisioned.

## DNA-Stabilized Fluorescent AgNCs

### Synthesis Method and Principle

**Figure 1:**
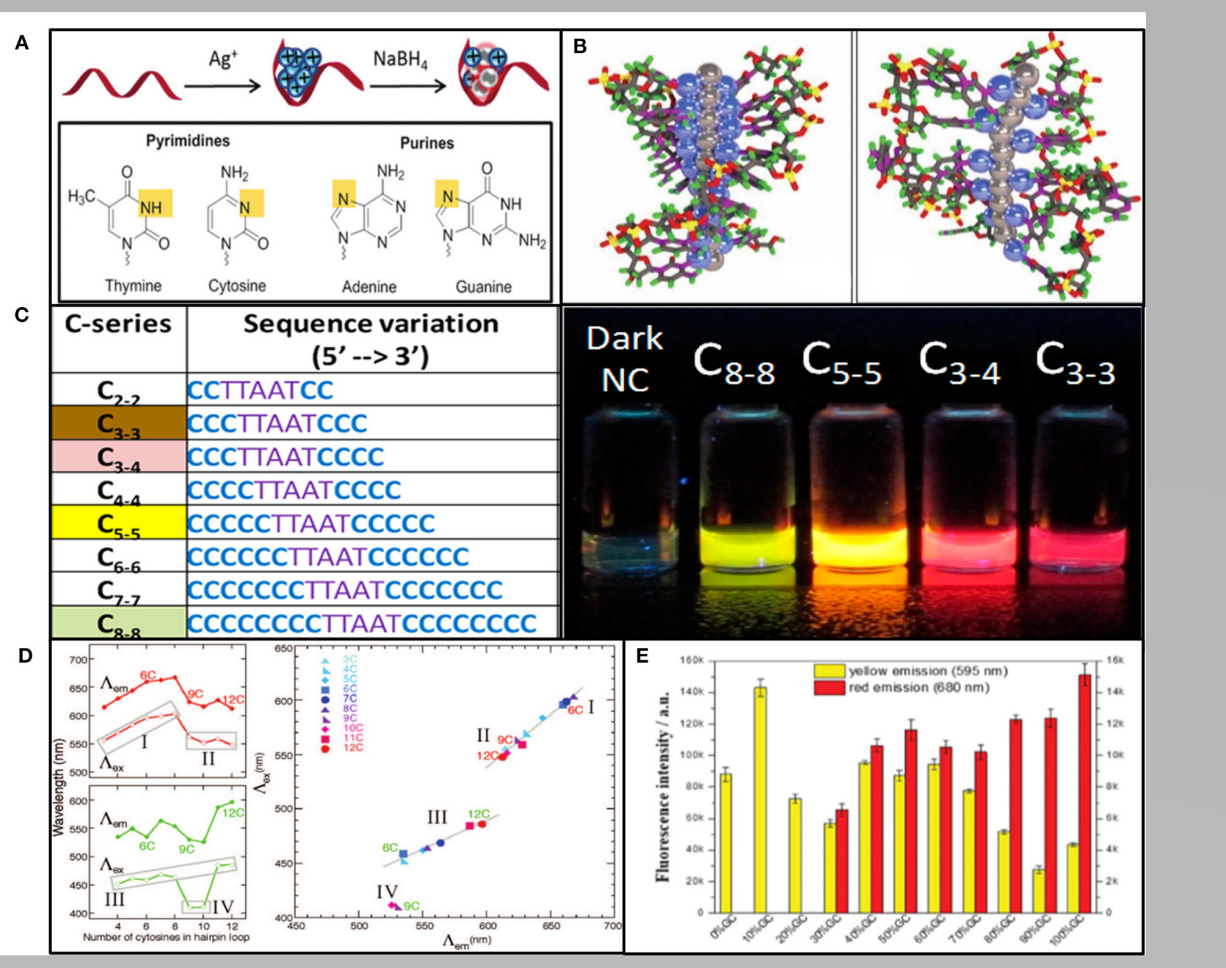
**(A)** The red band, blue balls and gray balls representing the ssDNA template, Ag^+^, and Ag^0^, respectively. Pyrimidine N_3_ and purine N_7_ interacting with Ag^+^ are shown in yellow (New et al., 2016). **(B)** The rod-like model of the AgNCs with neutral Ag atoms represented by gray balls and Ag^+^ cations represented by blue balls in the poly(C) DNA. Left: in repeat tetramer units. Right: in repeat trimer units (Schultz et al., 2013). **(C)** Varying the length of the C base leads to AgNCs varying in color (Obliosca et al., 2014). **(D)** The number of C in the hairpin ring determining the wavelength (left) and the excitation vs. the emission wavelengths (right) (O'Neill et al., 2009). **(E)** The fluorescence intensities of the hairpin-AgNCs synthesized by different percentages of GC content in the stem sequences (Guo et al., 2020).

DNA-AgNCs was first synthesized by Dickson's group, displaying bright fluorescence in a specific solution, which have attracted increasing research interest over the past decade (Petty et al., 2004). The DNA used to construct AgNCs can be single-stranded DNA, double-stranded DNA, triple-stranded DNA or even DNA nanostructures (Ma et al., 2011; Feng et al., 2012; O'Neill et al., 2012). The synthesis of DNA-AgNCs requires silver salt, an oligonucleotide with a C-rich sequence, and a reducing reagent of which micromolar amounts must be mixed at an appropriate ratio. For a ssDNA template, the most commonly employed ratio of Ag^+^: DNA is 6:1 and excess Ag ^+^ may help to form plasmonic nanoparticles (Hua et al., 2015). Following the reduction step, the incubation time ranges from several minutes immediately after the addition to 2 days depending on the different DNA template, affinity of Ag-DNA, and even the temperature. It has been proven that Ag^+^ have a higher binding affinity to nucleic acid bases than phosphate residues, which binds to bases via N_3_ of the pyrimidines or N_7_ of the purines with an affinity order of cytosine (C) > guanine (G) > adenine(A) > thymine (T) (Arakawa et al., 2001; Petty et al., 2004; Zikich et al., 2010) (Figure 1A). Fluorescent DNA-AgNCs contain both Ag^+^ and Ag^0^ with Ag^+^ mediated binding to the bases (Schultz et al., 2013) (Figure 1B). Although the detailed process of DNA-AgNCs formation is unclear, sufficient evidence proved that the cluster structure of fluorescent DNA-AgNCs is rod-shaped with free electron core (Schultz et al., 2013).

### Factors Affecting the Fluorescence of DNA-AgNCs

The mechanism of fluorescence origin is generally considered to be through the intra brand electron transition in the AgNCs (visible excitation), as well as a charge transfer between the DNA base and the AgNCs (UV excitation). Although the specific mechanism of the energy transfer from UV excited state to visible (or NIR) emission state is not clear, the fluorescent DNA-AgNCs are generally excited via the DNA bases (O'Neill et al., 2011). Therefore, DNA was the primary factor affecting the emissive properties of AgNCs, while the number of Ag, environmental factors (such as pH, metal ions, and macromolecular polymer) can also influence fluorescent emission (Kennedy et al., 2012; Schultz and Gwinn, 2012; Huang et al., 2013; Cerretani and Vosch, 2019).

The color of AgNCs could be adjusted to green, yellow, red and NIR by changing the position and number of clusters in the nucleation sequence (Obliosca et al., 2014) (Figure 1C). Later, Copp et al. discovered certain DNA based patterns or “motifs” correlating to AgNCs with similar fluorescence spectra. Dark motifs are much more abundant in T according with the exceedingly weak T-Ag^+^ association. G-rich motifs are most common in the red clusters, while C-rich motifs are related to all the bright color classes and dominating in the red class (Copp et al., 2018). Moreover, Weadick and Liu showed (2015) poly(T) only provided fluoresced green-emitting AgNCs at high pH. The specific wavelength of the AgNCs fluorescence also depended on the interaction between DNA secondary or tertiary structures.

There are two main fluorescence products in different length C-ring hairpins, which are green-emitting and red-emitting (O'Neill et al., 2009) (Figure 1D). A comprehensive study has been conducted regarding the effect of stem sequences on the fluorescent properties of hairpin-AgNCs. Guo et al. reported that the fluorescence intensity increased with the elongation of stem length when the content and distribution of GC remain unchanged. The Yellow hairpin AgNCs are generally formed in weak acid environment, and the weak alkali is conducive to the formation of red hairpin AgNCs (Guo et al., 2020) (Figure 1E). In addition, the relationship between the structural and fluorescence emission of DNA-AgNCs was also studied. Recently, Shah et al. (2020) reported that the orange emitting AgNCs were localized on the interface via a head-to-head binding of two DNA hairpins, while green, red, or NIR emissive AgNCs were embedded in a hairpin and double stranded DNA templates. The hairpin DNA can convert into G-quadruplex (G_4_) or i-motif structures, while the G_4_-AgNCs only showed intense peaks and the i-motif AgNCs exhibited poor peaks (Li et al., 2011). This result demonstrated that DNA structural changes can remarkably influence the spectral behavior of AgNCs.

Besides DNA sequences and structures, the number of Ag (N_Ag_) is another crucial factor. Visible-NIR emitting DNA-AgNCs can be premised on the N_Ag_ ~10–20 atom cluster centers. Larger Ag clusters tend toward longer λ_em_ since they provide more surface sites to connect higher numbers of bases. Although dark species appear at lower N_Ag_, by itself, N_Ag_ does not control whether DNA-AgNCs fluoresce (Schultz and Gwinn, 2012). On the other hand, it is more convenient to modulate the fluorescence emission of AgNCs by ion than by DNA sequence and length. Ions (such as Cu^2+^ and Hg ^+^) can quench the fluorescence of AgNCs by promoting the formation of non-fluorescent complexes (Guo et al., 2009; Zhang and Ye, 2011). Contrarily, the more stable AgNCs under the action of Zn^2+^ can increase the fluorescence intensity (Zhang et al., 2016). Additionally, hairpin DNA can be transformed into G_4_ or i-motif structure in the presence of K ^+^ and H ^+^, which affects the spectral behavior of AgNCs (Li et al., 2011).

## Methods Used for Fluorescent Signal Output

In recent years, nucleic acids (especially DNA) have stood out as excellent building blocks for nano-construction due to the programmability and specificity of the Watson-Crick base pairing (Chen et al., 2015; Li et al., 2018d). For DNA-templated nanomaterials, DNA molecules are first used as templates during the synthesis of nanomaterials, after which it can also be utilized as recognition units to avoid subsequent functional modifications (Qing et al., 2020). DNA-functionalized nanomaterials offer the ability to directly address the desired targets while enhancing the utility of these components during sensing and imaging (Pandya et al., 2016; Tian et al., 2020). DNA can selectively bind to a variety of analytes, including small organic molecules, ions, peptides, proteins and cells. This review summarized the progress based on the signal mechanism because a variety of signal methods have emerged to detect many different analytes.

### The Fluorescence Signal Is Based Directly on DNA-AgNCs

**Figure 2:**
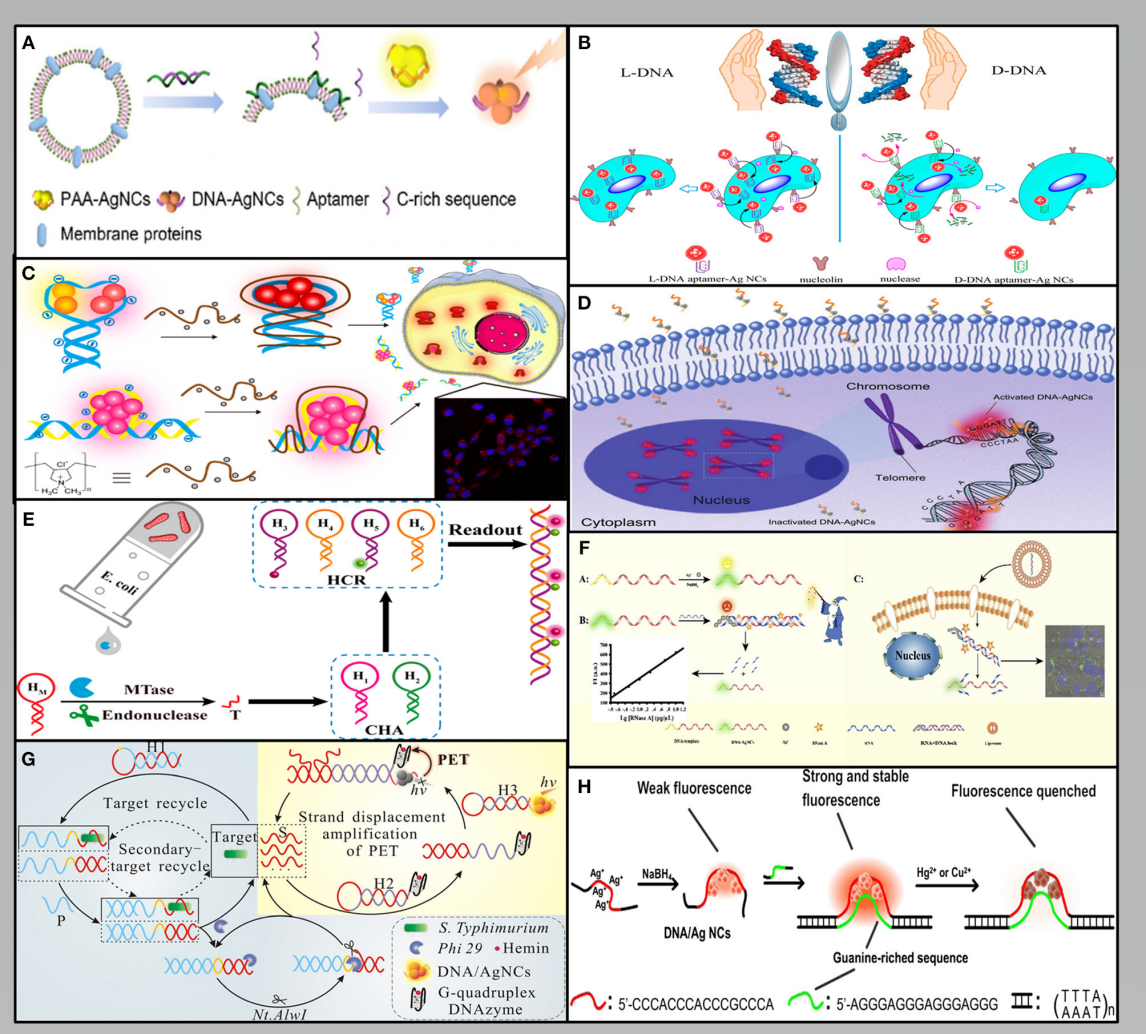
Methods used for fluorescence signal output: **(A–C)** The fluorescence signal is based directly on DNA-AgNCs (Han et al., 2016; Liu et al., 2017; Lyu et al., 2019). **(D–F)** The fluorescence signal is based directly on DNA-AgNCs (Huang et al., 2018; Li et al., 2018a; Dang et al., 2020). **(G)** The fluorescence signal is based on PET (Leng et al., 2018). **(H)** The fluorescence signal is based on reactive quenchers (Peng et al., 2015).

The specific DNA sequence is essential for the preparation of fluorescent AgNCs and detection. Aptamer-functionalized AgNCs as a target detection or imaging system has attracted increasing research attention. For example, Liu et al. designed a duplex that can release single-stranded C-rich sequences and the aptamer, sgc8 when it binds to the protein tyrosine kinase-7 (PTK7) on the cell membrane. Furthermore, these sequences can bind and transfer the AgNCs from the poly (acrylic acid) (PAA) matrix to amplify the fluorescence emission. The expression of PTK7 in a single HeLa cell and a CCRF-CEM cell was 7.5 × 10 ^−19^ mol and 1.8 × 10 ^−18^ mol, respectively (Liu et al., 2017) (Figure 2A). Cao et al. used a single labeling platform allowing for non-invasive fluorescence imaging and amplified electrochemical detection. The detection limit is three cells, which provides a biocompatibility and high specificity method for fully evaluating cancer cells (Cao et al., 2019b). Han et al. (2016) demonstrated that the L-conformation of DNA (L-DNA) could also be used to prepare aptamer-AgNCs, whose extraordinary resistance to nuclease digestion gives it higher biological stability and allows cell type specific imaging at physiological temperatures (Figure 2B). Lyu et al. modified fluorescent DNA-AgNCs with poly dimethyl diallyl ammonium chloride (PDDA) to make them interact with negatively charged phosphate groups in DNA chain. Moreover, rapid cell imaging can be performed in NIH/3T3 cells, because PDDA modification can significantly extend the stability of AgNCs and increase the cellular uptake of DNA-AgNCs (Lyu et al., 2019) (Figure 2C).

In addition, the color of the fluorescence-emission species can easily be transformed due to their susceptibility to oxidation. Therefore, unique small molecules displaying oxidative activity can be measured using this ability of AgNCs. Kathryn et al. found that that the fluorescence emission at 610 nm of AgNCs reversibly changed to 530 nm in an oxidized environment, which can be used to detect H_2_O_2_, the byproduct of the reaction between glucose and glucose oxidase. This strategy can measure glucose concentrations up to 20 mM, just within the glucose levels found in the blood (Schroeder et al., 2019). Li et al. found the rolling-circle-amplification-stabilized AgNCs provided a promising fluorescent probe for the detection of reactive oxygen species/nitrogen species (ROS/RNS) in living cells, because the hydrogel environment contributes to the stability of clusters. The detection limit was determined to be 58 nM, which was similar to the sensitivity of other fluorescent probes reported recently (Li et al., 2018c).

### The Fluorescence Signal Based on DNA Strand Hybridization

DNA stands out as an excellent material in biological system due to its predictable base-pair interactions and its programmable sequences (Tan et al., 2014). Many detection methods have been carried out based on the proximity of G that can induce the reversible conversion between dark species and a bright red-emitting species (Yeh et al., 2010). Huang et al. took advantage of the telomerase elongation product, the repetitive TTAGGG sequences to realize the detection of telomerase activity. The repetitive sequences hybridized with their complementary sequences led to a significant enhancement of the fluorescence because the G-rich sequence is close to AgNCs. The telomerase activity of an HeLa cell was calculated to be 1.2 × 10^−11^ IU in the exponential phase (Huang et al., 2018) (Figure 2D). Another interesting sensor depend on the structural changes caused by DNA hybridization. Li et al. developed a facile and specific cell nucleus imaging method based on the fluorescence activation of C-rich dark AgNCs by G-rich telomeres, and strong signal response could be achieved within 5 min (Li et al., 2018b).

As an important factor affecting the emission of DNA-AgNCs, the modulation of secondary DNA structures was used in the ratio detection system (Shah et al., 2018). Xu et al. developed a proportional visual analysis sensor platform for miRNA-21 in cells based on the self-assembly of AgNCs triggered by chain displacement. The detection limit of miRNA can reach picomole. Furthermore, based on the unlabeled sensing platform, the visual differentiation between cells can be realized according to the fluorescence color (Xu et al., 2020b). Li et al. reported that the upstream catalytic hairpin assembly (CHA) circuit can continuously produce a DNA product, which can be used to activate the downstream hybrid chain reaction (HCR) circuit to produce a significantly amplified fluorescence signal. This CHA-HCR amplifier makes it possible to detect M.SssI selectively and sensitively and the detection limit is 1.2 × 10^−4^ U/mL (Li et al., 2018a) (Figure 2E). Dang et al. designed an RNA strand that was applied to quench the fluorescence of DNA-AgNCs through the formation of RNA/DNA duplex. Moreover, the fluorescence signal of the AgNCs was restored after the degradation of RNA by RNase A. The fluorescence signal is linearly positively correlated with the concentration of RNase A, and the detection limit is 0.098 pg/μL (Dang et al., 2020) (Figure 2F).

### The Fluorescence Signal Based on Energy Transfer

The output of fluorescence signal mainly depends on the photophysical mechanism based on fluorescence resonance energy transfer (FRET) and surface plasma enhanced energy transfer (SPEET), and photoinduced electron transfer (PET). FRET can change the intensity of fluorescence emission by transferring energy from one dye molecule to another dye molecule (Chen et al., 2012). Since the fluorescence of AgNCs can be quenched by CNPs oxide, a new type FRET sensor was successfully constructed to detect human immunodeficiency virus (HIV) DNA sequence. The results showed that HIV-DNA could be detected in the range of 1–50 nM and the detection limit was 0.40 nM (Ye et al., 2016). Similar to FRET, SPEET belongs to the dipole surface interaction between the molecular and metal nanoparticle owing to the large volume ratio of the nano-metal surface (Yun et al., 2005). A method for sequence-specific DNA detection was developed based on the SPEET process between the DNA-AgNCs and gold nanoparticles (AuNPs). The DNA-AgNCs spontaneously adsorbed on the surface of AuNPs, while the AuNPs serve as a “nano-quencher” to quench the fluorescence of the DNA-AgNCs. The method achieved a detection limit of ~2.5 nM (Ma et al., 2015). Alternatively, by utilizing the PET mechanism, the signal can be modulated by the separation or closed between G_4_ DNAzyme and AgNCs (Zhang et al., 2013). Leng et al. described a fluorometric strategy for the ultrasensitive and highly specific detection of pathogenic bacteria. This strategy was dependent on a combination of target modulated photoinduced electron transfer (PET) and cyclic exponential amplification of hairpin probes. The biosensor system has high selectivity to *S. Typhimurium* with a detection limit of 8 cfu·mL^−1^ (Leng et al., 2018) (Figure 2G).

### The Fluorescence Signal Based on Reactive Quenchers

The fluorescence of AgNCs can be modulated not only by DNA hybridization, but also by interaction with environmental factors, such as thiolated compounds, and heavy-metal ions. For example, cysteine (Cys) can quench the fluorescence of DNA-AgNCs could be quenched by Cys via the specific interaction of robust Ag-S bonds, thus developing a label-free method for detecting Cys with the detection limit of 0.05 nm (Wang et al., 2018). Peng et al. designed a new fluorescent double-stranded DNA-AgNCs probe for the sensitive detection of Hg^2+^ and Cu^2+^. Both ions could effectively quench the emission of DNA-AgNCs that can be detected within detection limits of 2.1 and 3.4 nM, respectively (Peng et al., 2015) (Figure 2H). Zhang et al. constructed a DNA-AgNCs based biosensing system to achieve the rapid and sensitive detection of D-enantiomers (DAAs), which is specifically relevant in gastric cancer. The signal output was realized by fluorescence quenching in the presence of H_2_O_2_ and •OH, which were produced by the metabolism of DAAs. The detection limit is within the effective range of DAA concentration in the early stage of gastric cancer, which indicates that this method may be used in the early diagnosis of gastric cancer (Zhang et al., 2017).

## Conclusion

DNA-AgNCs, as promising label-free fluorophores, have the advantages of small size, low toxicity, adjustable fluorescence characteristics, and easy functional assembly, making them good candidates for fluorescent probing. Various applications have already been reported for detecting different targets (e.g., nucleic acids, small molecules, proteins, metal ions, germ, and cells) and cell imaging. To summarize the current research, analyzing the target via the fluorescent “on-off” mechanism can be achieved in the following four kinds of ways: using the fluorescence of the AgNCs directly; using strand hybridization (structural transformation or G-rich proximity) to increase or decrease the fluorescence; using electron transfer to realize a change in the fluorescence signal; and quenching the fluorescence using a reactive quencher. These methods fully demonstrate the significant application potential of AgNCs as fluorescent signals in analytical detection.

Although there has been exciting progress in the development of DNA-AgNCs, there are still many challenges to be solved in the future, such as the relationship between the structural characteristics and fluorescence emission, as well as the detailed formation process of cluster nucleation and aspects of their composition, which hinders the applications of DNA-AgNCs to some extent. The application of DNA-AgNCs *in vivo* systems remain limited due to the stability of the complex environments, and the toxicity in living cells. Therefore, a systematic and comprehensive assessment of their toxicity and stability in cells and animals is required before it can be considered as a reliable competitor to the standard protein fluorescence systems used today. Furthermore, a rational design involving fluorescent AgNCs capable of multicolored emissions during multiplexed detection and imaging is invaluable for their potential use. More importantly, DNA-AgNCs may require new design structures or the introduction of chemical modifications to obtain the desired properties.

## Author Contributions

MY, XC, YS, HL, and HZ wrote the article. XL and WX conceived, revised, and reviewed the article. All authors contributed to the article and approved the submitted version.

## Conflict of Interest

The authors declare that the research was conducted in the absence of any commercial or financial relationships that could be construed as a potential conflict of interest.
